# Cross‐species transmission of deltacoronavirus and the origin of porcine deltacoronavirus

**DOI:** 10.1111/eva.12997

**Published:** 2020-07-31

**Authors:** Xu Ye, Yingjin Chen, Xinyu Zhu, Jiahui Guo, Da Xie, Zhenzhen Hou, Shangen Xu, Junwei Zhou, Liurong Fang, Dang Wang, Shaobo Xiao

**Affiliations:** ^1^ State Key Laboratory of Agricultural Microbiology College of Veterinary Medicine Huazhong Agricultural University Wuhan China; ^2^ The Key Laboratory of Preventive Veterinary Medicine in Hubei Province Cooperative Innovation Center for Sustainable Pig Production Wuhan China

**Keywords:** cross‐species transmission, deltacoronavirus, molecular evolution, porcine deltacoronavirus

## Abstract

Deltacoronavirus is the last identified *Coronaviridae* subfamily genus. Differing from other coronavirus (CoV) genera, which mainly infect birds or mammals, deltacoronaviruses (δ‐CoVs) reportedly infect both animal types. Recent studies show that a novel δ‐CoV, porcine deltacoronavirus (PDCoV), can also infect calves and chickens with the potential to infect humans, raising the possibility of cross‐species transmission of δ‐CoVs. Here, we explored the deep phylogenetic history and cross‐species transmission of δ‐CoVs. Virus–host cophylogenetic analyses showed that δ‐CoVs have undergone frequent host switches in birds, and sparrows may serve as the unappreciated hubs for avian to mammal transmission. Our molecular clock analyses show that PDCoV possibly originated in Southeast Asia in the 1990s and that the PDCoV cluster shares a common ancestor with Sparrow‐CoV of around 1,810. Our findings contribute valuable insights into the diversification, evolution, and interspecies transmission of δ‐CoVs and the origin of PDCoV, providing a model for exploring the relationships of δ‐CoVs in birds and mammals.

## 1 INTRODUCTION

Coronaviruses (CoVs) are important pathogens that can cause serious, fatal and highly epidemic diseases in humans and other animals. Since the outbreak of severe acute respiratory syndrome CoV (SARS‐CoV) in 2003 and Middle East respiratory syndrome CoV (MERS‐CoV) in 2012, CoVs have attracted more and more attention (De Groot et al., 2013; Peiris, Yuen, Osterhaus, & Stohr, 2003). Deltacoronaviruses (δ‐CoVs) were first detected in wild Asian leopards and Chinese ferret badgers in 2006 during virological surveillance in southern China (Dong et al., 2007), and are also a newly identified genus in the *Coronaviridae* subfamily. Subsequently, three avian δ‐CoV species were reported in 2009 and seven other δ‐CoV species were identified in birds or pigs between 2009 and 2012, respectively (Woo et al., 2012). In 2014, an emerging swine enteropathogenic coronavirus, a porcine deltacoronavirus (PDCoV), was detected in the United States (Wang, Byrum, & Zhang, 2014). PDCoV has now been widely reported in the United States and Asia, where it has caused huge economic losses in farming. Recent researches have shown that δ‐CoVs are present in birds such as pigeon, falcon, and quail in Saudi Arabia (Lau et al., 2018), and sparrow in the United States (Chen et al., 2019); the PDCoVs are present in Tibetan pigs (Wang et al., 2018).

Host range is defined as the number of host species infected by a virus, which is a virus trait to understand the epidemiology and pathogenicity of pathogens. As one of the major factors in viral evolution, viruses rarely spread effectively in new hosts that were previously unexposed or uninfected (Lau & Chan, 2015). The cross‐species transmission of the virus poses a continuing threat to public health. Due to the increased contact between humans and other animal species, there is the possibility of cross‐species transmission and subsequent disease outbreaks (Parrish et al., 2008). Among RNA viruses, CoVs contain the large RNA genomes, ranging from 26 to 32 kilobases in length, which make them genetically more permissive to genome modification. About two‐thirds of each CoV genome comprises two overlapping ORFs (Orf1a and Orf1ab). The remaining genome includes the structural proteins' ORFs, namely Spike (S), envelope (E), membrane (M), and nuclear protein (N). Differences also exist in the accessory proteins encoded by different CoVs (Gorbalenya, Luis, John, & Snijder, 2006). Previous studies have reported that cross‐species transmission of CoVs is closely associated with the S protein, the nonstructural protein 3 (nsp3) (papain‐like protease, PLpro), and the accessory protein(s) (Cui, Li, & Shi, 2019; Forni, Cagliani, Clerici, & Sironi, 2017; Forni et al., 2016).

Coronaviruses have a large host spectrum, through mutation and recombination; CoVs have an increased probability of interspecies host jumping and of novel CoVs emerging under specific conditions (Su et al., 2016). SARS‐CoV and recently emerged MERS‐CoV epidemics have proven the ability of coronaviruses to cross‐species barriers and emerge rapidly in humans. Presently, the greatest genetic diversity in α‐ and β‐CoVs is documented in bats, and previous studies have confirmed SARS‐CoV and MERS‐CoV are originated from bats (Hu et al., 2017; Memish et al., 2013); however, the evolutionary history of δ‐CoVs is poorly understood. Genetic diversity levels in δ‐CoVs suggest that they are likely to be widely distributed in avian species around the world. Even if no δ‐CoV has been found in human so far, we could not exclude that the host range of δ‐CoV (avian, pigs, and leopard cats) might preclude of cross‐species potential to other hosts, including humans. Therefore, the possible zoonotic spread of new viruses to humans would pose a significant threat to global public health. Here, we investigated the origin and evolution of δ‐CoVs. The data we have obtained will help with the preparation of countermeasures against the possible future risk of zoonotic transmission of δ‐CoV to humans.

## 2 MATERIALS AND METHODS

### 2.1 Phylogenetic and sequence distance analyses

Briefly, the complete genomes and selected primary genes (Orf1ab, S, E‐M‐N) from δ‐CoVs were aligned using MAFFT (Katoh & Standley, 2013). Maximum‐likelihood (ML) phylogenetic trees were constructed using IQ‐TREE (Nguyen, Schmidt, von Haeseler, & Minh, 2015), and the best‐fitting nucleotide substitution model was determined automatically by the program following 1,000 bootstrap replicates. The results were visualized using iTOL v.4 (http://itol.embl.de/). The sequence distance analyses were assessed by the *SSE* v1.3 package (Simmonds, 2012), and sequence divergence scans were performed for the different viral hosts and generated by the inbuilt Sequence Distance program.

### 2.2 Virus–host evolutionary relationships

To examine virus–host co‐divergence in δ‐CoVs, we performed cophylogenetic reconstructions using Jane (program version 4) (Conow, Fielder, Ovadia, & Libeskind‐Hadas, 2010). The phylogenetic tree was based on the ML tree, and the host topology was obtained from the TIMETREE website (http://www.timetree.org/). We used different event costs to test the result, and the number of generations and population size were both set to 100. Four cost schemes were tested: 0‐1‐1‐1‐1 (Aiewsakun & Katzourakis, 2017), 0‐1‐2‐1‐1 (Jane's default setting), 0‐1‐1‐2‐0 (Conow et al., 2010), and ‐1‐0‐0‐0‐0 (Xu, Zhao, Gong, & Han, 2018).

### 2.3 Recombinant analyses

Nucleotide sequence similarity analyses were assessed by SimPlot v.3.5.1 (Lole et al., 1999) (sliding window size, 500 bp; step size, 100 nucleotides; 1,000 bootstrap replicates), and results were visualized using GraphPad Prism software v.6.01.

### 2.4 Codon usage analyses

Cross‐species transmission in viruses can involve a change in codon preference, which allows the virus to adapt to the new host and support self‐proliferation (Bahir, Fromer, Prat, & Linial, 2009). Genomic GC content is one of the most reliable signals relating to cross‐species codon usage variation (Chen, William, Hottes, Lucy, & Mcadams, 2004), and wobble in the third position (the GC3s) of the codons may indicate viral evolution (Bera et al., 2017). The nucleotide contents (GC, GC12s, and GC3s) of each δ‐CoVs coding sequence were calculated using the Galaxy website (https://galaxy.pasteur.fr) and CondonW software. GC contents were plotted against GC12s and GC3s using Graphpad Prism v6.01. The codon usage heatmap was drawn by TBtools (Chen, Xia, Chen, & He, 2020), and as there was no significant difference in the codon usage among the PDCoVs, we listed all the PDCoVs as a group. The frequently used codons with higher relative synonymous codon usage values were represented by the largest red circle, the medium frequently used codons by a smaller circle and the lower frequency codon usage by a large green circle.

### 2.5 Bayesian evolutionary analysis of PDCoVs

To better understand the relationship between the Sp‐CoVs and PDCoVs, the time for viral origin was explored through the time‐scaled phylogenetic tree constructed in BEAST 2 using the standard Yule Model (Bouckaert et al., 2014). To further compare the time of origin for the different genotypes of the PDCoV isolates, a Bayesian time‐scaled tree and phylogeographic tree for the PDCoVs were prepared in BEAST 2 using the Mascot package (Muller, Rasmussen, & Stadler, 2018). This algorithm entirely avoids migration history sampling. The genomic dataset was analyzed with a strict clock under a single GTR + gamma substitution model. The states were sampled every 10,000 steps, and 10% of samples were discarded during burn‐in with an MCMC chain length of 100 million. The parameters in the result were checked by estimating the effective sample sizes with Tracer 1.7 (Rambaut, Drummond, Xie, Baele, & Suchard, 2018) and visualized using Figtree v1.4.3 (tree.bio.ed.ac.uk/software/figtree/).

### 2.6 Accessory protein analyses

VGAS (http://cefg.uestc.cn/vgas/) was used to search for potential protein‐encoding segments in deltacoronaviruses and visualized them using IBS v1.0 (Liu et al, 2015). We arranged the viruses according to the ML phylogenetic tree, and the similarity of PLpro, RNA‐dependent RNA polymerase (RDRP), S and some accessory proteins was compared with Dabbling Duck‐CoV and PDCoV.

## 3 RESULTS

### 3.1 Phylogenetic analyses and genetic divergence of δ‐CoVs

**Figure 1:**
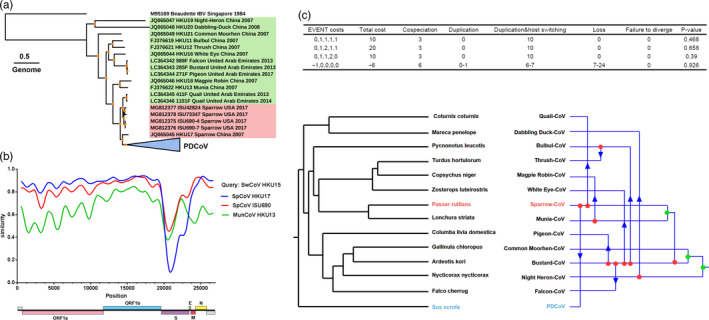
Phylogenetic and host–virus evolutionary analyses for δ‐CoVs. (a) ML phylogenetic tree for the δ‐CoVs genome. The best‐fitting nucleotide substitution model was determined automatically by the program following 1,000 bootstrap replicates, and the phylogenetic trees were visualized using iTOL v.4 (Interactive Tree of Life, http://itol.embl.de/). The PDCoVs collapsed into one node are shown in a blue triangle, the Sparrow‐CoV is shown in red font, and the other ADCoVs are shown in green font; bootstrap support values higher than 95 are shown with orange dots**.** The sequence names of δ‐CoVs are shown in a uniform format (NCBI accession number‐Strain name‐Target host‐Isolated country‐Isolated year). (b) Genome‐based recombination analysis using SimPlot v3.5. The settings were as follows: window size, 500; step size 20; gap stripping, on; Kimura distance model. (c) The different event costs used for the host–virus phylogeny congruence test. All possible cospeciation, duplication, and host switch events are shown. Sp‐CoV is marked in red font. PDCoV is marked in blue font

To characterize the genetic diversity of δ‐CoVs among different hosts, we constructed a phylogenetic tree based on the 118 complete δ‐CoVs genomes. The sequences of 18 avian deltacoronaviruses (ADCoVs) and 100 PDCoVs were obtained from the National Center for Biotechnology Information (NCBI, https://www.ncbi.nlm.nih.gov) for analyzing phylogenetic and host–virus evolutionary relationships (Table S1). Using the genome from infectious bronchitis virus (IBV, a γ‐CoV genus member) as the outgroup, maximum‐likelihood (ML) phylogenetic trees were constructed based on the complete genome (Figure 1a) from δ‐CoVs using IQ‐TREE. Based on the phylogenetic tree topologies and viral hosts, the δ‐CoVs separated into three groups: PDCoVs, Sp‐CoVs, and other bird‐CoVs. Moreover, we constructed three phylogenetic trees based on the ORF1ab, S, and E‐M‐N genes sequences of δ‐CoVs. The topological structure is basically consistent between genome and ORF1ab; however, Sp‐CoVs strains showed inconsistent topology in the phylogenetic trees of S and E‐M‐N gene, differing from the ORF1ab gene phylogenetic tree (Figure S1a). This inconsistent topologies of S and E‐M‐N genes of Sp‐CoVs, in which outlier sequences were found in other bird‐CoVs subgroups in a phylogenetic tree (Figure S1a), might be attributed to cross‐species transmission or/and genomic recombination.

To further explore the evolution of δ‐CoVs, phylogenetic analysis of the complete genome and S gene among the *Coronaviridae* subfamily shows that the complete genomes of δ‐CoVs share a close kinship with those of other γ‐CoVs, while the S gene from δ‐CoVs shares a closed kinship with those from other α‐CoVs (Figure S2, Table S2). Moreover, the intra‐ and inter‐group genetic distance (p‐distance) analyses were used to further quantify genetic divergence of δ‐CoVs. As shown in Figure 1b,c the δ‐CoVs shared a higher sequence similarity in ORF1ab and E‐M‐N genes. In contrast, a considerable genetic diversity is shown in the S gene. Moreover, the genetic diversity in ADCoV is generally higher than that in PDCoV (Figure S1b). Interestingly, ML‐trees and p‐distance analyses showed that for all genes Sp‐CoV is closest to PDCoV, suggesting a strong correlation between these viruses (Figure 1a and Figure S1).

### 3.2 Host–virus evolutionary relationships for δ‐CoVs

Sequence divergence for primary genes and the p‐distances of the intra‐ and inter‐groups and the synonymous and nonsynonymous p‐distances were calculated. The results showed that PLpro, the S gene, and the accessory proteins from the δ‐CoVs displayed high levels of molecular variability (Figure S1b and Figure S3). Interestingly, the fluctuation range for the synonymous and nonsynonymous mutation rates showed that the S gene from Sp‐CoVs had a large span through all δ‐CoVs. Moreover, our sequence similarity and recombination analyses showed that the newly discovered Sp‐CoV (GenBank accession number MG812375) is more similar to PDCoV across the whole genome (Figure 1b), suggesting that the origin of PDCoV may have occurred via recombination between different Sp‐CoVs. A statistically significant signal for phylogenetic incongruence in δ‐CoVs showed that PDCoV might evolved from a recombination event, with the 5′ part of the S gene acquired from one Sp‐CoV (ISU690 isolate) and the remaining genomic regions acquired from other Sp‐CoV (HKU17 isolate) (Figure 1b).

To further discern the spread of δ‐CoVs among different hosts, we performed event‐based cophylogenetic reconstructions using the Jane program (version 4), because it can analyze five types of events in a host–virus phylogeny (cospeciation, duplication, duplication and host switching, loss and failure to diverge) with each event having a related cost. The different event costs produced the same results in that there are large amounts of host switching in δ‐CoVs (Figure 1c), suggesting that Sp‐CoV might have switched its host from sparrow to swine, thereby generating PDCoV.

The PLpro and S genes from PDCoV were further analyzed to evaluate adaptive evolution in this virus by looking for sites where positive selection may have occurred using PAML (Yang, 2007). There are 2 sites showing evidence of positive selection in PLpro gene and 12 sites under positive selection in S gene, which are mainly located in N‐terminal domain of S gene (S1‐NTD) (Figure S4). We also have listed all the potential accessory proteins from δ‐CoVs according to the ML phylogenetic tree, and our results reveal that the 3′‐tail sequence of PDCoV is shorter than in other δ‐CoVs (Figure S3).

### 3.3 Codon usage bias in δ‐CoVs

**Figure 2:**
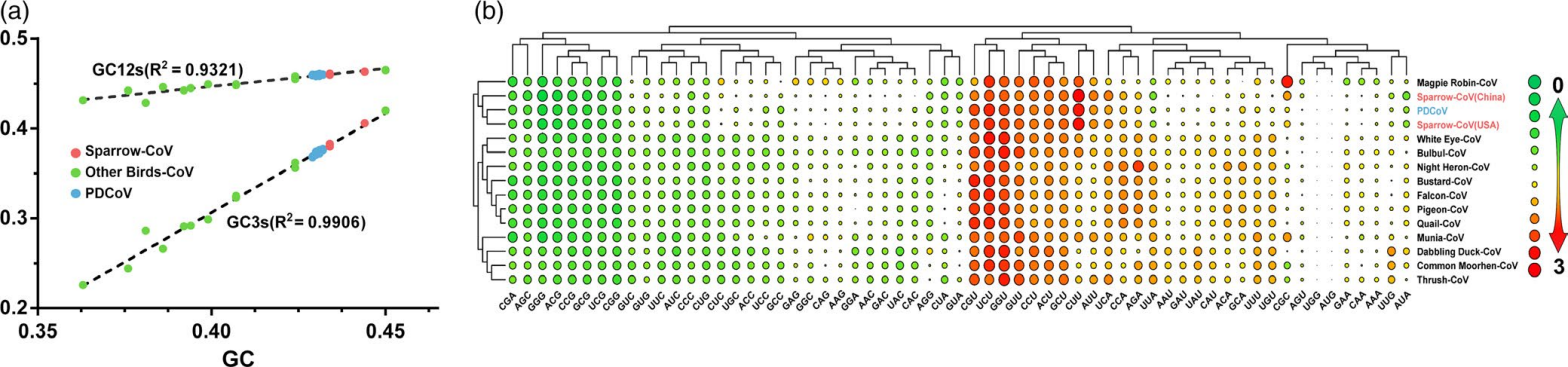
Analysis of codon bias in the primary genes from δ‐CoVs. (a) GC % plotted against GC12s and GC3s. The *x*‐axis represents GC content. The GC12 and GC3 contents correspond to the values on the *y*‐axis. (b) Relative synonymous codon usage (RSCU) comparisons between different δ‐CoV hosts. As PDCoVs have similar codon usage, the data were normalized for analysis. Frequently used codons with higher RSCU values are represented by the largest red circle, medium frequently used codons are represented by a smaller circle, while the lower frequency used codons are represented by a larger green circle

Codon usage bias plays an indispensable role in viral evolution. Our analysis showed that the total GC content of δ‐CoVs ranged from 0.35 to 0.48, the GC12s (average GC content in the first and second codon positions) ranged from 0.4 to 0.5, and the GC3s ranged from 0.21 to 0.42 (Figure 2a). Changes in GC content are mainly caused by GC3s, and there was a strong correlation between the different types of δ‐CoVs in the change of GC content with GC12s (*R* ^2^ = .9321) and GC3s (*R* ^2^ = .9906), further confirming that the δ‐CoVs had undergone long‐term evolution and that PDCoV might come from ADCoV.

In addition, the codon usage biases between distinct ADCoVs and PDCoVs were calculated and grouped using hierarchical, complete linkage clustering. The outcome of the cluster analysis was shown as the heatmap based on the RSCU value (green—low frequency and red—high frequency). The heatmap showed high similarity in the codon usage of PDCoVs and Sp‐CoVs (Figure 2b). The codon usage of PDCoVs and Sp‐CoVs differed from that of other avian CoVs, suggesting that the PDCoVs had kept the previous codon preference and had not yet fully adapted to the new host (Figure 2b). The genetic similarity, codon usage, and GC bias results imply that perhaps PDCoVs originated from ADCoVs, especially the Sp‐CoVs.

### 3.4 Origin and evolution of PDCoVs

**Figure 3:**
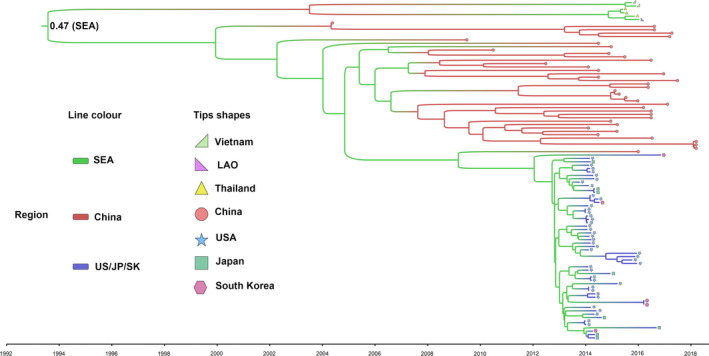
Maximum‐clade‐credibility tree showing ancestral time and locations for the PDCoVs inferred from the structured coalescent. The branch colors indicate the location states, and the tip shapes of the tree represent the isolated countries of the PDCoVs, as shown in the regional legend. The node at the root of the tree represents the root state posteriori probability of the SEA

To better understand the potential evolutionary origin of PDCoVs, the time‐scaled phylogenetic tree of PDCoVs was constructed in BEAST 2. As shown in Figure S5a, the time of origin for the PDCoVs was around the 1990s, while the time point for the Sp‐CoVs was much earlier, supporting the speculation that the PDCoVs originated from the Sp‐CoVs. A new ML phylogenetic tree was conducted to explore the genotypes of the PDCoVs. Based on this tree, the PDCoVs separated into three major lineages: Southeast Asia (SEA) (Thailand, Vietnam and Laos People's Democratic Republic, *n* = 6), China (*n* = 39) and USA group, and South Korea and Japan (*n* = 55), separately (Figure S5b). This indicates that genetic diversity in worldwide PDCoVs is geographically distributed. Moreover, the Bayesian time‐scaled tree of PDCoVs indicated that PDCoVs might have originated around 1993 (1992–1995, 95% highest posterior density). The root of the phylogeny was inferred to be most likely in SEA (root state posterior probability = .47, Figure 3), and SEA might be a source location mainly for strains in South Korea, Japan, and the United States.

## 4 DISCUSSION

In recent years, CoVs such as SARS‐CoV and MERS‐CoV, which originated in animals, have had seriously effects on public health. Between November 2002 and July 2003, SARS caused an eventual 8,098 cases, resulting in 774 deaths reported in 37 countries. At the end of September 2019, a total of 2,468 laboratory‐confirmed cases of MERS, including 851 deaths, were reported globally (Park, Park, Song, How, & Jung, 2019). Current research shows that α‐CoVs and β‐CoV only infect mammals, whereas γ‐CoVs mainly infect birds. For δ‐CoVs, most of them infected with avian and some other members could infect mammals, indicating that they have undergone host‐switching events between these animals. Here, our results reveal that the S protein from δ‐CoVs showed a close relationship with those from other α‐CoVs. Because the S protein plays an essential role in CoV entry, this protein in δ‐CoVs may determine whether viral infection in birds or mammals is successful, making successful viruses a potential threat if their mammalian host range expands.

Currently, δ‐CoVs have been detected in many birds, and substantial genetic differences occur among different viral species. Moreover, genetic diversity in ADCoV exceeds that in PDCoV, suggesting a long evolution of δ‐CoVs in birds. Thus, a possible risk is that δ‐CoVs will spread more widely in birds. As we know, Southeast Asia contains one of the most globally abundant and diverse bird populations, and previous research indicates that avian influenza always originates in Asia, especially Southeast Asia (Martin et al., 2006). Whether a high risk for new ADCoV infections in mammals exists in Southeast Asia deserves future attention. Certainly, the close genetic similarity, codon usage, and GC bias in δ‐CoVs isolated from sparrows and pigs make the Sp‐CoV/PDCoV lineage particularly attractive for researchers in the context of cross‐species transmission. S gene from Sp‐CoVs had a large span through all δ‐CoVs, which may allow the virus to spill over into new hosts. The Munia‐CoV and PDCoV S genes are highly similar, prompting speculation that PDCoV may have arisen from a recombination event between Sp‐CoV and Munia‐CoV (Lau et al., 2018). However, our results suggest that the origin of PDCoV may be the result of a recombination between different Sp‐CoVs. Interestingly, the GC content of PDCoVs is slightly below that of Sp‐CoVs, possibly promoting adaptation in the avian‐derived virus to replicate in mammals. Wong, et al. reported that the selective pressures on human influenza virus reduced the GC content of the viral genome, and this might assist the formation of less stable viral mRNA structures at lower host temperatures and enable the virus to escape the innate immune system (Wong, Smith, Rabadan, Peiris, & Poon, 2010). It is possible that a similar situation would enable PDCoV to undergo efficient replication in pigs via a reduced GC content. Additionally, δ‐CoVs are prone to cross‐species transmission in birds, but the lower interspecies hurdles in pigs would make them more susceptible to avian virus infections. Surprisingly, the PDCoV CHN‐AH‐2004 isolate (GenBank accession number: KP757890) from preserved Chinese fecal samples collected in 2004 shares a similar origin as the PDCoVs detected from Tibetan pigs in 2016–2017 (Dong et al., 2015), suggesting that PDCoVs existed and spread in China a long time ago.

Generally, cross‐species transmission of CoV is closely associated with the viral S protein, PLpro, and the accessory proteins. As one of the main structural proteins, their S protein participates in receptor‐binding and host adaptability (Cui et al., 2019). PLpro is involved in processing the viral polyproteins and regulates the innate immune response (Forni et al., 2016). Our results showed that more sites underwent positive selection in the PLpro gene than in the S gene (2 sites versus 12 sites), which implies that the S gene has played a more important role during PDCoV evolution. For the accessory proteins, δ‐CoVs own open reading frames (ORFs) encode a wide variety of accessory proteins, some of which are host derived, while some have been lost during viral evolution. Studies have shown that the accessory proteins in PDCoV target the host's antiviral innate immune responses, which are also thought to promote viral adaptation to the host (Fang et al., 2018). Our results reveal that the number of accessory proteins in PDCoV is fewer than in the other δ‐CoVs. This suggests that some of the accessory genes are not essential for PDCoV replication in pigs and are also not the most important factor for the host switch from birds to mammals.

In summary, our analyses provide in‐depth insights into the diversification, evolution, and interspecies transmission of δ‐CoVs and the origin of PDCoV. Increasing evidence strongly implicates wild birds as the reservoir hosts for δ‐CoVs, though transmission of the virus within bird populations remains unknown. Given that the birds like sparrows share the ecological niche with domestic mammals, sparrows might act as a potential intermediate host, which play a role in transmission of δ‐CoVs to pigs. Following initial pig infection, the pig‐to‐pig transmission is a predominant feature of PDCoV outbreaks. Given that pigs are in frequent contact with human and wild animals, the lower interspecies hurdles in pigs would make them a potential mixing vessel for δ‐CoVs. Thus, there is still a risk that δ‐CoVs may spread to more mammals, including human. Although sparrows are suspected to be the primary source of infection in pigs and the δ‐CoV genomes from pigs and sparrows are highly similar, the routes of direct or indirect interspecies transmission are yet unknown. Therefore, detailed case–control studies are needed to unravel the exact transmission routes.

## CONFLICT OF INTEREST

None declared.

## Supporting information

Figure S1

Figure S2

Figure S3

Figure S4

Figure S5

Table S1

Table S2

Appendix S1

## Data Availability

The data that support the findings of this study are available in the Appendix S1 of this article.
