# Supplementary figures and images for: Cross‐species transmission of deltacoronavirus and the origin of porcine deltacoronavirus
Source: Evol Appl. 2020 Jul 31;13(9):2246–53. doi: 10.1111/eva.12997 (PMC7273114; doi:10.1111/eva.12997)

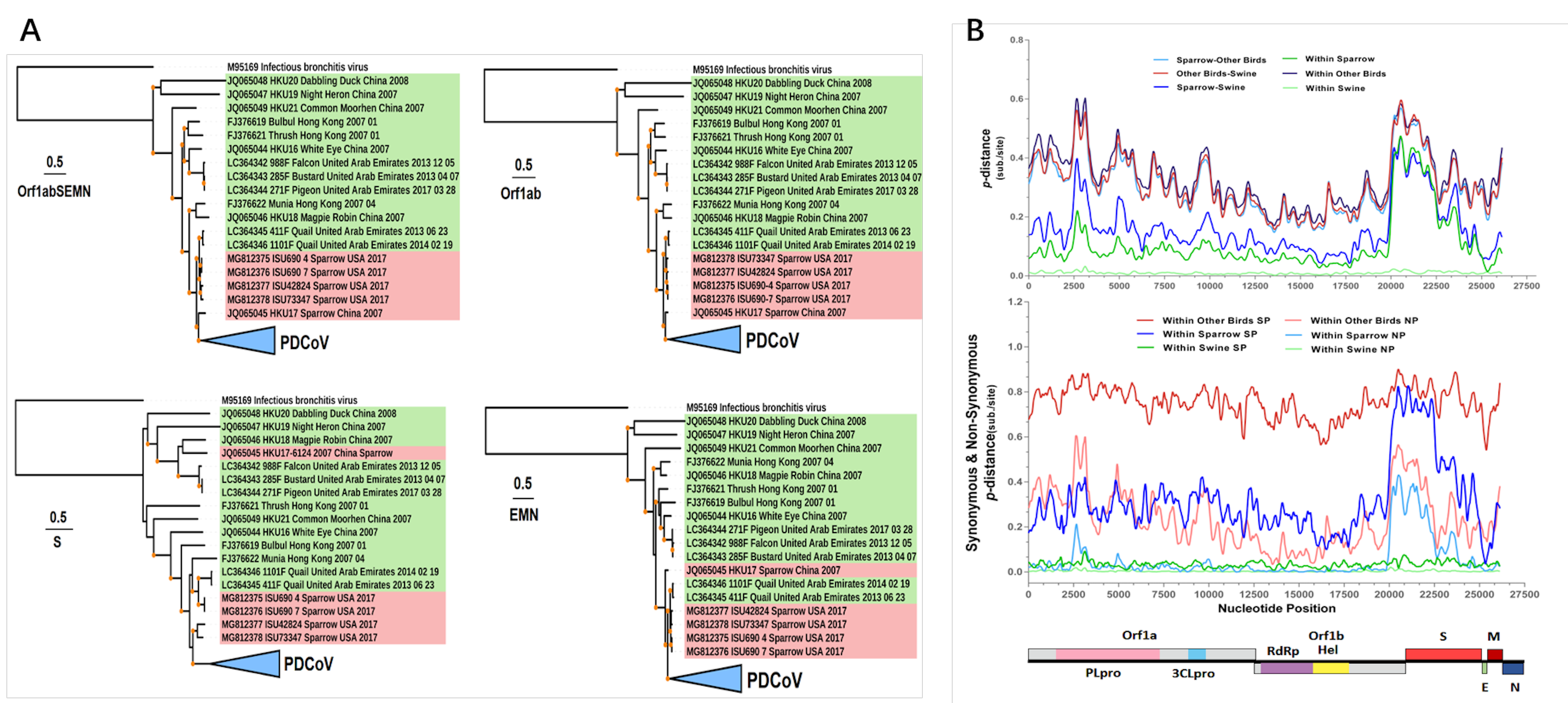

Supplement: Supplementary file 1 — Figure S1 [file EVA-13-2246-s001.tiff]

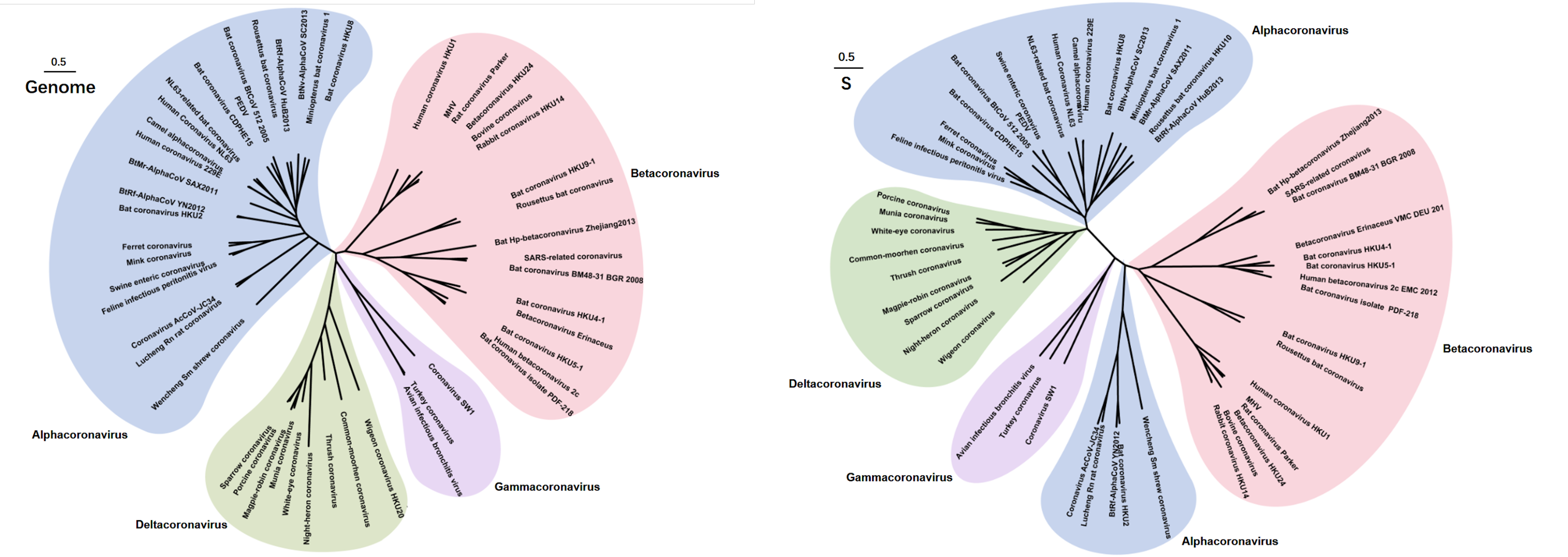

Supplement: Supplementary file 2 — Figure S2 [file EVA-13-2246-s002.tiff]

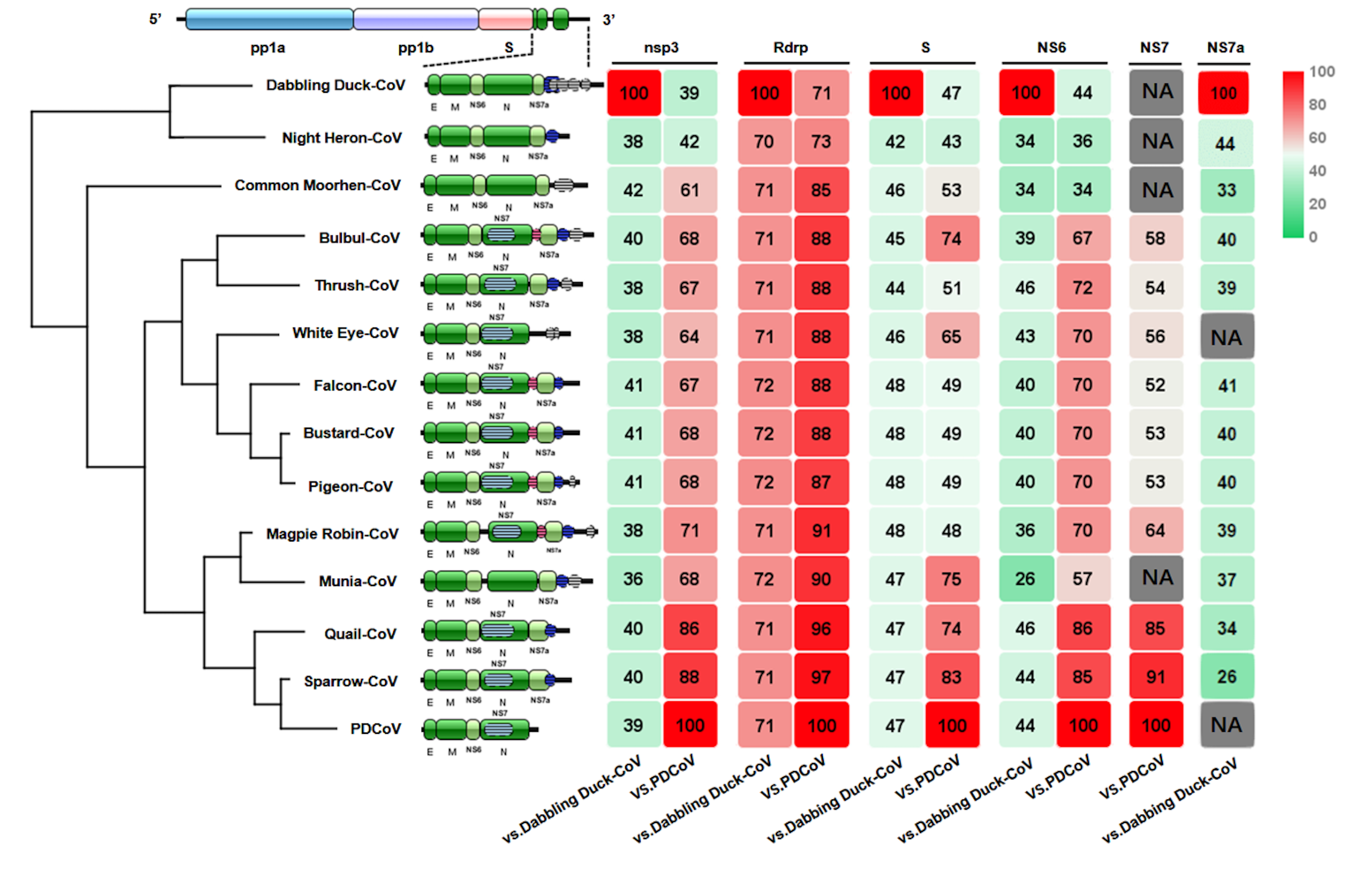

Supplement: Supplementary file 3 — Figure S3 [file EVA-13-2246-s003.tiff]

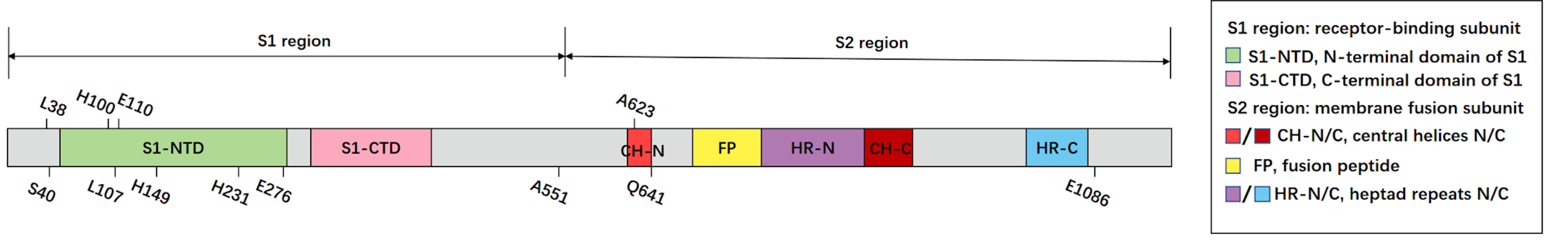

Supplement: Supplementary file 4 — Figure S4 [file EVA-13-2246-s004.tiff]

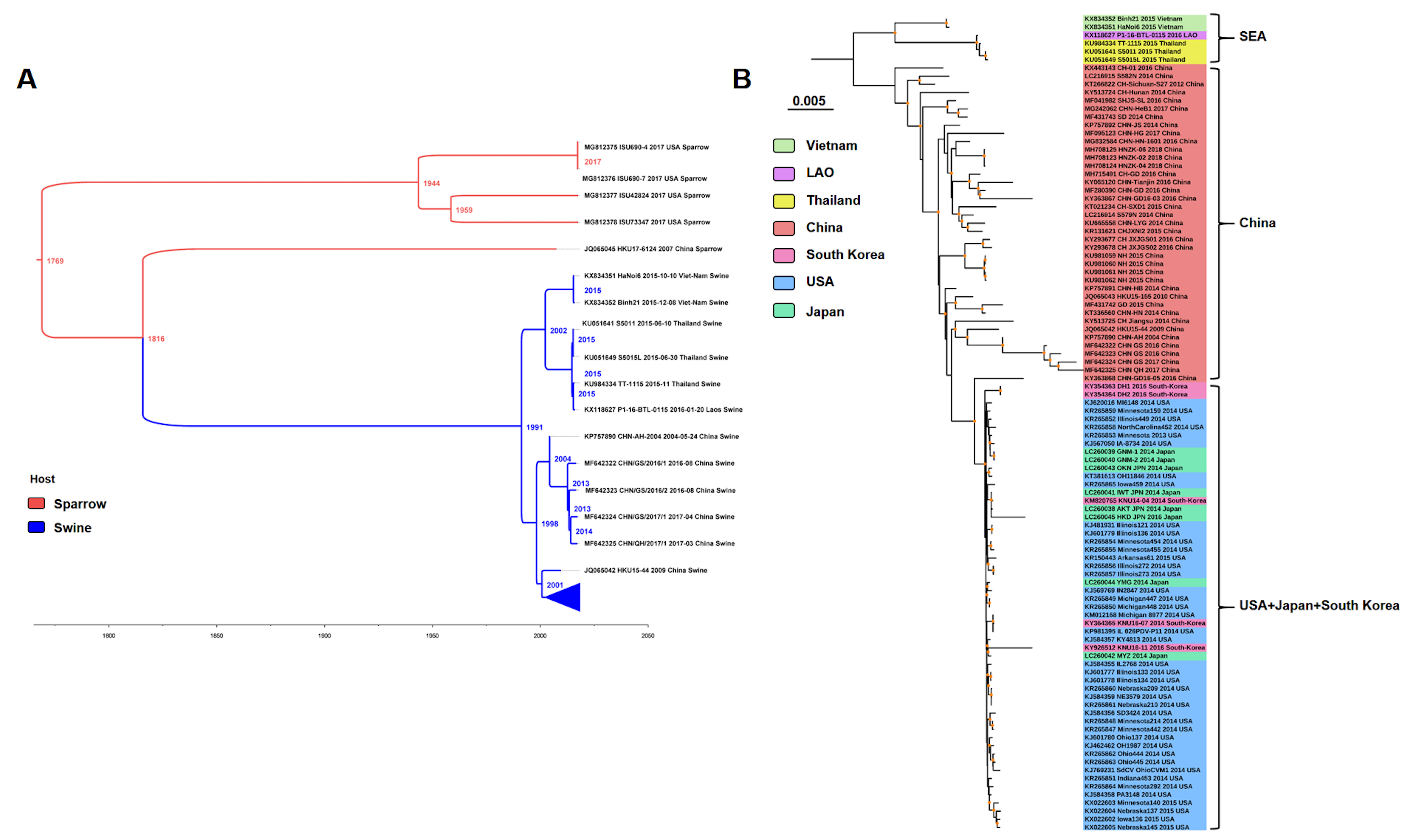

Supplement: Supplementary file 5 — Figure S5 [file EVA-13-2246-s005.tiff]
