# Supplementary material for: Cross‐species transmission of deltacoronavirus and the origin of porcine deltacoronavirus
Source: Evol Appl. 2020 Jul 31;13(9):2246–53. doi: 10.1111/eva.12997 (PMC7273114; doi:10.1111/eva.12997)
Supplement: Supplementary file 8 — Appendix S1 [file EVA-13-2246-s008.docx]

# Supplementary Materials

# Cross-Species Transmission of Deltacoronavirus and the Origin of Porcine Deltacoronavirus

**Figure S1. Phylogenetic and sequence distance analyses of δ-CoVs. (A)** Estimated ML trees are shown for concatenated ORF1ab-S-E-M-N, ORF1ab, S, concatenated E-M-N genes of δ-CoVs. The PDCoVs were collapsed into one node, and infectious bronchitis virus (IBV, a γ-CoV genus member) as an outgroup. Intra- and inter-group genetic distances (p-distance) are shown in the inset, bootstrap support values higher than 95 are shown with brown dots. **(B)** The nucleotide sequence divergence (p-distance, synonymous and non-synonymous) between δ-CoVs intra- and inter-groups were used for comparison, using 249-nt fragments by 25-nt increments across the aligned sequences.

**Figure S2. Phylogeny of the CoVs.** The phylogenetic trees of complete genome sequences and S gene were constructed using IQ-TREE, and the α-CoVs are indicated by blue, β-CoVs in pink, γ-CoVs in purple, δ-CoVs in green.

**Figure S3. The potential accessory proteins of δ-CoVs.** Genomic structure of δ-CoV with potential protein-encoding segments using alignment data paired with Maximum-likelihood phylogenetic tree. Heat maps were constructed by the degree of genetic similarity of nsp3 (PLpro), RDRP, S and a set of representative accessory proteins of δ-CoVs.

**Figure S4. The positive selection sites on S gene of PDCoVs.** The numbering refers to the position of the site in JQ065043.

**Figure S5. Bayesian time-scaled and Maximum-likelihood phylogenetic tree of PDCoVs. (A)** Bayesian time-scaled tree of Sp-CoVs and PDCoVs. The branch colors indicate the host, and the original time is listed on the node. The PDCoV isolates were collapsed into one node are shown in a blue triangle. **(B)** Maximum-likelihood phylogenetic tree analyses of the PDCoVs, the label colors indicate the region, bootstrap support values higher than 95 are shown with brown dots.
